# Carotid Wallstent placement difficulties encountered in carotid artery stenting

**DOI:** 10.1186/2193-1801-2-468

**Published:** 2013-09-16

**Authors:** Kaoru Myouchin, Katsutoshi Takayama, Toshiaki Taoka, Hiroyuki Nakagawa, Takeshi Wada, Masahiko Sakamoto, Satoru Iwasaki, Shinichiro Kurokawa, Kimihiko Kichikawa

**Affiliations:** Department of Radiology and Interventional Neuroradiology, Ishinkai Yao General Hospital, 1-41 Numa, Yao, Osaka, 581-0036 Japan; Department of Radiology, Nara Medical University, 840 Shijo-cho, Kashihara, Nara, 634-8522 Japan; Department of Radiology, Nara Prefectural Nara Hospital, 1-38-1 Hiramatu, Nara, 631-0846 Japan; Department of Radiology, Higashiosaka City General Hospital, Nishiiwata 3-4-5, Higashiosaka, Osaka, 578-8588 Japan; Department of Neurosurgery, Ishinkai Yao General Hospital, 1-41 Numa, Yao, Osaka, 581-0036 Japan

**Keywords:** Carotid artery stenting, Carotid Wallstent, Inflection point, Lesion tortuosity

## Abstract

**Purpose:**

The present study aimed to identify the types of curved lesions that are difficult to place Carotid Wallstent (CWS).

**Materials and methods:**

The study targeted 31 consecutive carotid artery (CA) stenosis underwent carotid artery stenting using CWS. CWS placement success rate, stenosis location, lesion tortuosity, and relationship with stent placement failures were investigated. Lesion tortuosity was defined as the angle formed by 2 tangential lines between internal CA and common CA from the inflection point (IP) was defined as the center of lesion curvature. Stenosed lesions were classified into type A or B. Type A was defined as if the distal end of the stenosis was located proximal to the IP at a distance ≥0.5 of a vertebral body based on the posterior height of the 3rd vertebral body, otherwise was type B.

**Results:**

The stent placement success rate was 93.5% (29/31). The 2 unsuccessfully stented lesions, both lesions were significantly different from other lesions by having a lesion tortuosity less than 90° and by belonging to type B.

**Conclusion:**

Since CWS placement is difficult in patients with CA stenosis located close to the IP at a lesion tortuosity ≤90°, open-cell stents should be considered as an alternative.

## Introduction

Carotid artery stenting (CAS) is increasingly being indicated as an alternative to carotid endarterectomy (CEA) not only for patients at high risk for CEA, but also for those in the normal risk group (Brott et al. 2011). However, CAS is reportedly associated with a higher incidence of perioperative stroke than CEA, indicating the need to reduce this incidence (Mantese et al. 2010). The incidence of perioperative stroke also reportedly differs according to the type of stent used; since the closed-cell Carotid Wallstent (CWS (Boston Scientific, Natick, MA, USA)) has a low incidence, its use has been recommended (Jansen et al. 2009). However, shortcomings of the CWS include low radial force and potentially difficult placement in curved lesions due to a tendency towards poor wall apposition compared to open-cell stents. Therefore, the present study sought to determine the frequency of CWS placement difficulties and to identify the types of tortuous lesions that are difficult to stent.

## Methods

The study included 31 lesions (10 symptomatic; 21 asymptomatic; mean stenosis rate (NASCET method); 81%) in 30 consecutive patients (26 men, 4 women; age range 59-84 years; mean age 71.7 years) who underwent standard CAS using the FilterWire EZ (FWEZ (Boston Scientific, Natick, MA, USA)) and CWS from May to November 2011.

### CAS procedure

The CAS procedure was performed under local anesthesia by placing an 8-French (F) guiding catheter in the common carotid artery (CCA) via the femoral artery and inserting the FWEZ past the stenosed region before deploying the filter. After subsequent intravascular ultrasound (IVUS (Volcano Therapeutics, Rancho Cordova, California)), the lumen diameter of the stenosed region, the lumen diameter of a normal blood vessel of the internal carotid artery (ICA) or of the CCA proximal to the stenosed region, and the lumen diameter of a normal blood vessel of the ICA distal to the stenosed region were all measured. The stent was then placed after predilatation with a 3-mm or 4-mm balloon (predilatation was not performed in ≤60% stenosis). The stent was placed in the ICA alone if there was no stenosis in the CCA and ≥50% of the ICA stenosis was located 0.5 of a vertebral body distal to the carotid bifurcation (wherein a single vertebral body is regarded as the height of the body of the third vertebra). In all other situations, the stent was placed from the ICA to the CCA. The stent also was placed from a position >1 cm from the distal part of the stenosis no regard to tortuosity. After stent placement, conservative postdilatation at a balloon diameter ≤80% of the distal ICA lumen diameter was performed (even in lesions with 30-40% residual stenosis), and the FWEZ was recovered. All patients underwent standard CAS according to this procedure.

### Endpoints

**Figure 1:**
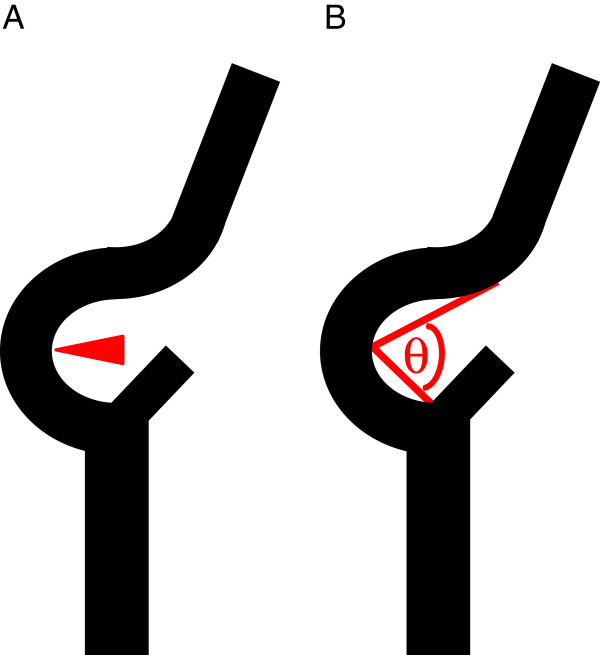
**Tortuosity and inflection point. A**. The inflection point is defined as the center of lesion curvature. **B**. Tortuosity is defined as the angle (θ) formed by 2 tangential lines drawn on the internal carotid artery side and the common carotid artery side starting from the inflection point.

**Figure 2:**
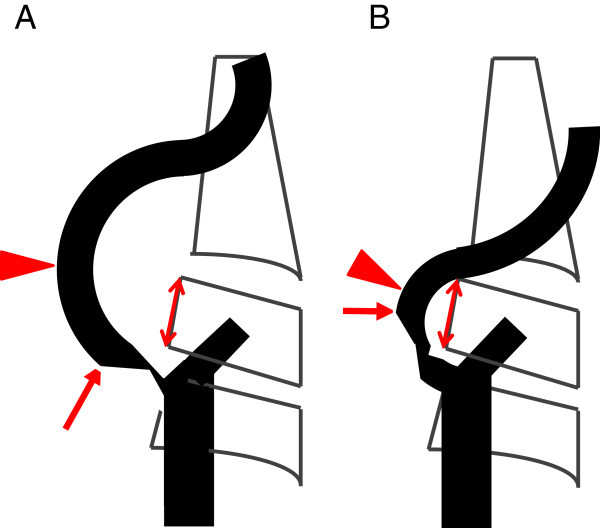
**Stenosis site and inflection point. A**. Type A lesion: lesion in which the distal end of the stenosis (arrow) is located proximal to the inflection point (arrowhead) at a distance ≥0.5 of a vertebral body based on the posterior height of the 3rd vertebral body (double arrow). **B**. Type B lesion: lesion in which the distal end (arrow) of the stenosis is located proximal to the inflection point (arrowhead) at a distance <0.5 of a vertebral body or located distal to the inflection point.

The CWS placement success rate and the association between stenosis location and tortuosity and stent placement failures were retrospectively investigated. Placement success was defined as the ability to place the stent via its distal portion at least 0.5 of a vertebral body from the distal end of the stenosis wherein a single vertebral body is equivalent to the height of the body of the third vertebra. The inflection point was defined as the center of lesion curvature. Lesion tortuosity was defined as the angle formed by the 2 tangential lines drawn on the ICA side and the CCA side starting from the inflection point, using the frontal or lateral common carotid arteriograms in which the curvature in the stenosis of the carotid artery was most visible (Figure 1). Stenosed lesions were classified into 2 types (Type A, B). Type A was defined as if the distal end of the stenosis was located proximal to the inflection point at a distance of ≥0.5 of a vertebral body, or type B if the distal end of the stenosis was located proximal to the inflection point at a distance of <0.5 of a vertebral body or if it was located distal to the inflection point (Figure 2).

### Statistical analysis

Continuous variables were compared using Mann-Whitney *U* test, and categorical variables were compared using the *χ*^2^ test. A p-value <0.05 was considered statistically significant.

## Results

**Figure 3:**
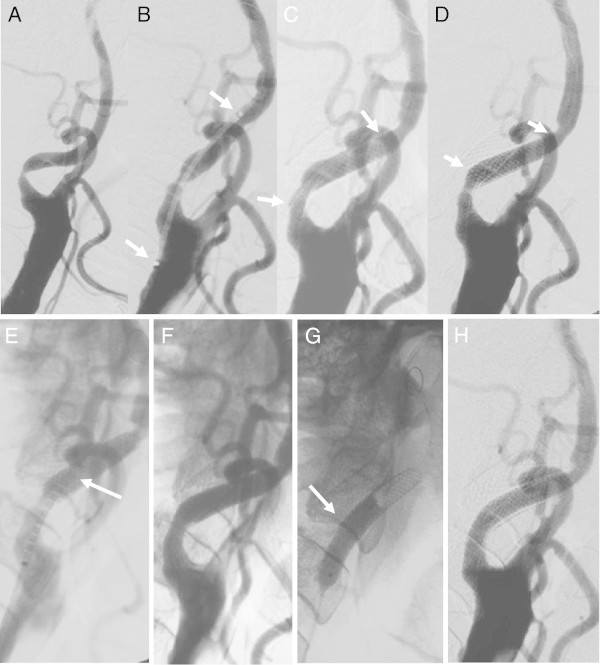
**Unsuccessful CWS placement (tortuosity 60°; type B lesion) (example 4).** Right common carotid arteriogram. **A**. Severe stenosis observed at the origin of the right internal carotid artery. **B**. Guiding the 6-mm × 22-mm CWS (arrow). **C**. Stent placement (arrow). **D**. Stent shortening and migration distally to the lesion observed one minutes after placement (arrow). **E**. Guiding the 6-mm × 20-mm precise stent to overlap slightly with the proximal end of the CWS (arrow). **F**. Precise stent deployment (arrow). **G**. Postdilatation with a 5-mm-diameter balloon (arrow). **H**. Good dilatation observed on the arteriogram taken immediately afterwards.

**Figure 4:**
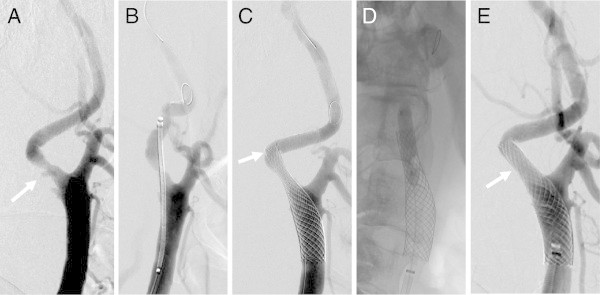
**Unsuccessful CWS placement (tortuosity 72.9°; type B lesion) (example 5).** Right common carotid arteriogram. **A**. Severe stenosis observed at the origin of the right internal carotid artery. **B**. Guiding the 10-mm × 24-mm CWS. **C**. Stent shortening proximally to the inflection point after placement (arrow). **D**. Postdilatation. **E**. Good dilatation observed on the arteriogram taken immediately afterwards.

**Figure 5:**
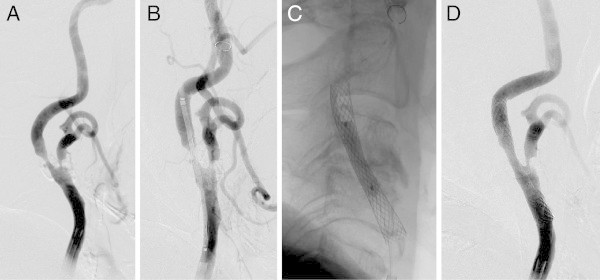
**Successful CWS placement (tortuosity 93.8°; type A lesion) (example 2)**. Right common carotid arteriogram. **A**. Severe 84% stenosis accompanied by mild tortuosity observed at the origin of the right internal carotid artery. **B**. Guiding the 10-mm × 24-mm CWS (arrow). **C**. Postdilatation. **D**. Good dilatation observed on the arteriogram taken immediately afterwards.

**Figure 6:**
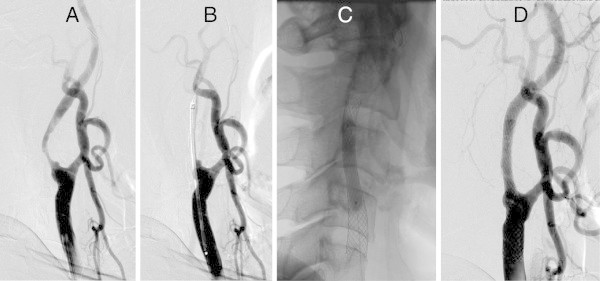
**Successful CWS placement (tortuosity 102.6°; type B lesion) (example 1).** Right common carotid arteriogram. **A**. Severe 99% stenosis accompanied by mild tortuosity observed at the origin of the right internal carotid artery. **B**. Guiding the 10-mm × 31-mm CWS (arrow). **C**. Postdilatation. **D**. Good dilatation observed on the arteriogram taken immediately afterwards.

**Figure 7:**
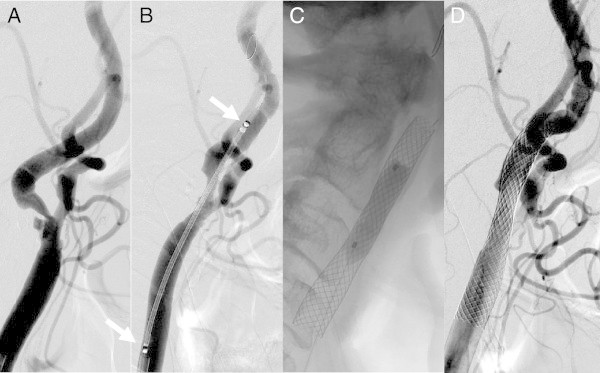
**Successful CWS placement (tortuosity 92.4°; type B lesion) (example 3).** Left common carotid arteriogram. **A**. Severe 80% stenosis accompanied by mild tortuosity observed at the origin of the left internal carotid artery. **B** Guiding the 8-mm × 21-mm CWS (arrow). **C**. Postdilatation. **D**. Good dilatation observed on the arteriogram taken immediately afterwards.

The placement success rate was 93.5% (29/31 lesions). Of the 2 lesions in which stent placement failed (6.5%) (Figures 3 and 4), a Precise stent (Cordis Endovascular, Miami Lakes, FL, USA) was placed in 1 lesion due to the inability to place the CWS in the optimal position (Figure 3). A minor postoperative stroke was observed in 1 of the 2 unsuccessful stent placement cases (3.2%), but the patient’s symptoms resolved completely within 2 weeks. Tortuosity ranged from 60° to 178.8° (mean 128.5°); 22 stenosed lesions were type A (Figure 5), while the remaining 9 lesions were type B (Figures 3, 4, 6 and 7). Tortuosity of the 2 unsuccessfully stented lesions, both of which were type B, was 60° and 72.9°, respectively. As such, both lesions were significantly different from other lesions for having a tortuosity <90° and for belonging to type B (Tables 1 and 2).

**Table 1:** **Stent placement success by tortuosity**

	<90°	≥90°
Stent placement success	0	29
Stent placement failure	2	0

P = 0.004.

**Table 2:** **Stent placement success by lesion type**

	Type A	Type B
Stent placement success	22	7
Stent placement failure	0	2

P = 0.022.

## Discussion

In a 2008 report from the Boston Scientific EPI: A Carotid Stenting Trial for High-Risk Surgical Patients (BEACH) study (Iyer et al. 2008), a multicenter prospective trial of 480 carotid artery stenosis patients at high risk for CEA in which CWS was used, the procedure success rate was 98.3%, and the failure rate was 1.7%. While the report did not provide detailed reasons for the failures, they may have been due to a high degree of tortuosity, as in the present study. Although stent placement difficulty occurred in 2 lesions (6.5%) in the present study, a different stent was eventually used in 1 of these lesions, so the stent placement failure rate was 3.75%. Both of these lesions had pronounced curvatures with tortuosity ≤90°.

A study by Lin et al. (2005) used tortuosity as an indicator of degree of curvature in CAS based on the angle of a central line running through the lumens of the CCA and the ICA, wherein an angle <30° was classified as mild, an angle of 30-60° was regarded as moderate, and an angle >60° was deemed severe tortuosity. The study found that moderate and severe tortuosities were significantly more prevalent in patients aged ≥80 years, suggesting that the frequency of severe carotid tortuosity is strongly influenced by patient characteristics. Similarly, 1 of the 2 unsuccessful stent placement patients in the present study was ≥ 80 years old (83 years old). However, while Lin et al.’s classification is an indicator of ICA curvature, it cannot be used as an indicator of curvature in CWS placement, because if the stenosed region is not located close to the inflection point, the stent can be placed without having to extend the curvature.

Care needs to be exercised when placing closed-cell stents because, unlike open-cell stents, they undergo shortening after placement and cause the stented blood vessel to straighten out. The manufacturer, Boston Scientific, therefore recommends placing the closed-cell CWS via its distal portion at least 1 cm from the distal end of the stenosed region. Delayed CWS shortening reportedly causes restenosis, because when a CWS is placed in a tortuous lesion, it causes straightening and shortening of the blood vessel, which in turn causes the stent to move caudally such that, particularly when there is a considerable difference between the lumen diameters of the ICA and CCA, shortening is likely to occur in the CCA, which has the bigger lumen diameter (Yoon et al. 2009). Takayama et al. (2011) argued that, in light of this delayed shortening, “longer is better” when selecting the length of a CWS. Since shortening must be taken into account when placing a CWS, in the present study, stent placement failure was defined as the inability to place the stent at least 0.5 of a vertebral body from the distal end of the stenosis. Both of the 2 unsuccessfully stented lesions were type B and had tortuosity ≤90°. Shortening occurred immediately after placement in both of these lesions, with the stent migrating to the distal part of the curve in 1 lesion and downwards to the proximal part of the curve in the other lesion. We think that this occurred because the inflection point was adjacent to the stenosis, so that placing the CWS caused the curve of the blood vessel to extend (straighten), thus preventing the stent from remaining in place.

The limitations of the present study were its small cohort size and retrospective design. Moreover, since the carotid arteries are fixed within the carotid duct, the blood vessels are less likely to expand as a result of CWS placement in the case of high lesions. The distance from the stenosed region to the entrance of the carotid duct may therefore be an influential factor, but this has yet to be investigated. We do believe, however, that evaluating the location of stenosed regions and the extent of tortuosity prior to surgery is a useful way to identify potential stent placement difficulties.

## Conclusion

Since Carotid WALLSTENT placement is difficult in patients with carotid artery stenosis located close to the inflection point at a tortuosity ≤90°, open-cell stents should be considered as an alternative.

### Ethics of this study

Any accompanying images and this study has done according to the guidance of Helsinki Declaration.
